# Diagnosis of COVID-19 Disease in Chest CT-Scan Images Based on Combination of Low-Level Texture Analysis and MobileNetV2 Features

**DOI:** 10.1155/2022/1658615

**Published:** 2022-11-30

**Authors:** Azita Yazdani, Shervan Fekri-Ershad, Saeed Jelvay

**Affiliations:** ^1^Department of Health Information Management, Clinical Education Research Centre, Health Human Resources Research Centre, School of Health Management and Information Sciences, Shiraz University of Medical Sciences, Shiraz, Iran; ^2^Faculty of Computer Engineering, Najafabad Branch, Islamic Azad University, Najafabad, Iran; ^3^Big Data Research Center, Najafabad Branch, Islamic Azad University, Najafabad, Iran; ^4^Department of Health Information Technology, Abadan University of Medical Sciences, Abadan, Iran

## Abstract

Since two years ago, the COVID-19 virus has spread strongly in the world and has killed more than 6 million people directly and has affected the lives of more than 500 million people. Early diagnosis of the virus can help to break the chain of transmission and reduce the death rate. In most cases, the virus spreads in the infected person's chest. Therefore, the analysis of a chest CT scan is one of the most efficient methods for diagnosing a patient. Until now, various methods have been presented to diagnose COVID-19 disease in chest CT-scan images. Most recent studies have proposed deep learning-based methods. But handcrafted features provide acceptable results in some studies too. In this paper, an innovative approach is proposed based on the combination of low-level and deep features. First of all, local neighborhood difference patterns are performed to extract handcrafted texture features. Next, deep features are extracted using MobileNetV2. Finally, a two-level decision-making algorithm is performed to improve the detection rate especially when the proposed decisions based on the two different feature set are not the same. The proposed approach is evaluated on a collected dataset of chest CT scan images from June 1, 2021, to December 20, 2021, of 238 cases in two groups of patient and healthy in different COVID-19 variants. The results show that the combination of texture and deep features can provide better performance than using each feature set separately. Results demonstrate that the proposed approach provides higher accuracy in comparison with some state-of-the-art methods in this scope.

## 1. Introduction

It has been more than two years since the beginning of the COVID-19 global epidemic. According to official figures from the World Health Organization (WHO), the number of people infected with the coronavirus has exceeded 500 million, and by May 2022, more than 6 million people have died from the virus [1]. The COVID-19 disease is not yet fully predictable in terms of clinical symptoms and shows significant potential for rapidly eroding healthcare [2] infrastructure. The inability to detect pandemics quickly remains a major challenge for health care systems worldwide [3]. The first and most important step in controlling this pandemic is to quickly identify patients and monitor for positive cases. Various diagnostic methods for rapid diagnosis of COVID-19 have been introduced by various studies and by the WHO [4]. Reverse transcription-polymerase chain reaction (RT-PCR) is the most prominent diagnostic method [4, 5]. Although the RT-PCR test is the standard reference for confirming COVID-19, in many studies, high false-negative rates have been reported as a limitation of this test [4–8]. RT-PCR test is also not sensitive enough in the early stages [7, 9]. Also, there is a contradiction between the lack of equipment for laboratory environments and the rapid and accurate screening of suspects. These limitations lead to delays in diagnosis, treatment, and increased mortality in the advanced stages of the disease [4].

In the early of the epidemic, when extensive PCR diagnostic tests were not available, medical imaging was often the only means of triaging patients suspected of having COVID-19. Despite the widespread distribution of PCR and other forms of serological testing, due to the stated limitations, medical imaging plays an important role in the diagnosis of this disease [3]. Medical imaging techniques, such as chest X-ray and computed tomography (CT) imaging of the chest, are considered competitive and complementary candidates for PCR tests [10]. Using CT images, radiologists can identify internal structures in more detail and evaluate their texture, density, size, and shape. Chest images obtained by CT scan usually provide much more accurate images of the patient's condition than X-rays [11].

Early detection of COVID-19 with high reliability is essential in the early stages. In existing studies, high detection speed and high sensitivity in the detection of COVID-19 by CT images compared to PCR have been reported [9]. Investing in infrastructure capable of maximizing data and information generated by action-based evidence for the detection, prediction, and management of COVID-19, including diagnostic imaging, is critical to optimizing resource utilization and guiding global strategy [12].

Artificial intelligence (AI) and machine vision techniques are one of the most effective infrastructures in medical imaging and have been very successful in the COVID-19 pandemic. Many researchers have used AI tools to differentiate COVID-19 from other conditions on chest CT. Many deep learning models claim to accurately diagnose COVID-19 using a chest CT and even distinguish it from pneumonia or other chest diseases [13]. Despite the high speed of diagnosis of COVID-19 disease using radiographic images compared to RT-PCR tests, manual examination of radiological images in pandemic conditions brings a lot of workload for physicians.

Timely diagnosis of COVID-19 from chest radiographs and severe dependence on the accuracy of COVID-19 diagnosis in the radiologist's opinion, which is often influenced by individual bias and clinical experience are the main reasons for using machine learning methods in detecting COVID-19. Deep learning-based methods are used to assist physicians in the rapid, accurate, and automated diagnosis of COVID-19 [14]. To reduce the enormous burden of reading radiological images for physicians and improve diagnostic accuracy, AI-based automated diagnostic systems are in high demand. These systems read X-ray or CT images of patients as input and present diagnostic results as output [7, 15]. Automated COVID-19 prediction models based on chest images are rapidly evolving to support medical decision-making. Since the outbreak of the coronavirus, many articles have been published on the automatic detection of COVID-19 through CT images.

**Figure 1:**
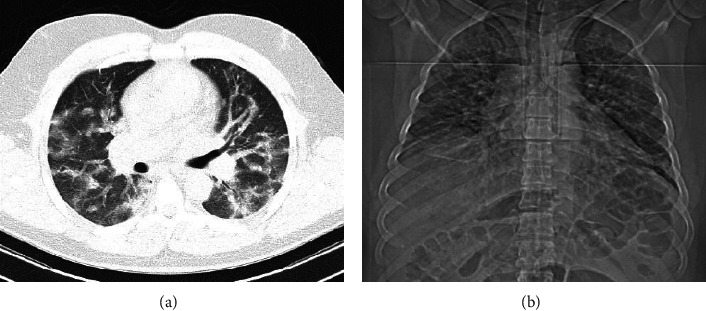
Chest CT-scan of a 51-years old male patient in two formats.

The main challenge in all the studies that have been presented so far is the accuracy of the classification process [16–23]. Most recent studies have proposed deep learning-based methods [16–20]. But, handcrafted features provide acceptable results in some studies too [21–23]. Researches show that lung texture in people who are infected with COVID-19 undergoes visible changes compared to healthy people. The chest CT-scan of a 51-years old male patient is shown in Figure 1 in two formats.

In this paper, an approach based on combining image texture features and high-level features such as deep feature maps for the detection of COVID-19 in chest CT images is proposed, called CTFDF for short. The distinguishing feature of this research from previous research studies is the use of a combination of low-level and high-level image features. Due to the obvious distinction between the chest texture of a patient and a healthy person, the local neighborhood difference patterns (LNDP) operator has been used to derive image texture features. These features are statistical and considered as low-level type because of their ability to easily interpret to the human vision system. High-level features have also been extracted using the MobilNetV2 deep network to increase classification accuracy. MobileNetV2 network has been considered in this paper due to its high speed and good accuracy in most medical applications. In the proposed approach, the fully connected layer has been removed from the MobileNetV2 structure and a flatten layer has been added to the end of the structure. Finally, for test samples that do not receive the same label based on the above two feature groups, an innovative two-level decision algorithm based on the similarity criterion is proposed.

In some previous studies, the MobileNetV2 has been used for various problems in the field of computer vision, and it has provided good results. The main novelty of this paper is the combination of image texture features along with features that are extracted from deep layers in the MobileNetV2. As mentioned above, some of the texture properties of the chest CT scan in Covid-19 patients have changes compared to healthy cases. Therefore, to extract this set of features and to increase the final accuracy of the diagnosis system, texture analysis operators such as LNDP can also be used. Also, in this paper, an innovative two-step algorithm is presented to determine the final label of the input image. Unlike many previous papers, the last layers in the MobileNetV2 have been removed and a supervised classifier has been used to diagnose patients. Therefore, in this paper, the combination of texture features and deep features is performed in the classification phase.

To evaluate the proposed approach, a dataset of chest CT scan images from June 1, 2021, to December 20, 2021, of 238 cases in two groups of patient and healthy are collected. All of the CT-scan images are collected in Ayatollah Taleghani Hospital Abadan, Iran. The patient samples were infected with COVID-19 in different COVID-19 variants, which is one of the main potentials of this study. The proposed approach is evaluated in terms of precision and accuracy rate. The results show that the combination of texture and deep features can provide better performance than using each feature set separately. Results demonstrate that the proposed approach provides higher accuracy in comparison with some state-of-the-art methods in this scope.

The rest of the manuscript is organized as follows.

In Section 2, a review of related research has been done. The proposed recognition approach is described in Section 3. In Section 4, the evaluation results of the proposed model are presented with the description of the used data set. The conclusion of the article is given in Section 5.

## 2. Related Works

In this section, some remarkable studies in this scope are described, which address one of the following topics: COVID-19 detection using handcrafted features or COVID-19 detection on chest X-ray or CT-scan images using deep learning. The main facts related to each of the related work are highlighted, such as the feature extraction phase, classification model, and type of image used in the experiments (CXR or CT).

Today, deep networks widely are used for image classification in different scopes. So, some researchers propose deep-based methods for COVID-19 classification based on chest CT-scan images. For example, Alshazly et al. proposed an advanced deep network architecture, in joint of a transfer learning technique for COVID-19 detection in chest CT scan images [15]. They employed different state-of-the-art deep networks, such as SqueezeNet, Inception, ResNet, ResNeXt, ShuffleNet, and DenseNet, as the backbone of the proposed approach. Also, to improve the final classification performance, the input image size is customized and tailored for each one of the deep architecture [15].

**Figure 2:**
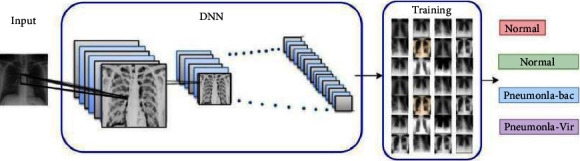
The structure of CoroNet [16].

In another research, Khan et al. proposed, a deep convolutional neural network (CNN) to detect COVID-19 infection from chest X-ray images titled CoroNet [16]. The CoroNet model is designed based on Xception architecture which is pretrained on the ImageNet dataset. Also, Khan et al., trained end-to-end their proposed CoroNet on a dataset prepared by collecting X-ray images such as COVID-19 and other chest pneumonia samples. The structure of their proposed network is shown in Figure 2. The CoroNet consists of more than 33 million parameters in 4 different layers such as Flatten, Dropout, Dense, and Dense-1. Reported results in [16] show that CoroNet provides a higher detection rate in comparison with VGG19, ResNet50, and DarkNet.

**Figure 3:**
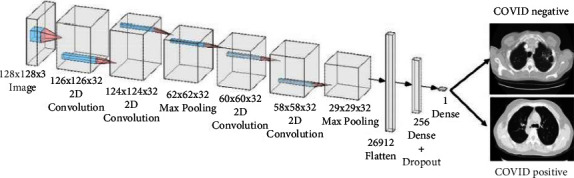
The structure of CTnet-10 proposed in [17].

Shah et al. [17] designed a CNN-based network called CTnet-10 to classify COVID-19 patients in lung CT-scan images. The CTnet-10 is fed with an input sample in size of 128 × 128 × 3. So, all of the input images should be resized, which can be considered as a limitation. There are a total of 4 convolutional blocks and two max-pooling sequential. Then, it is then passed through 26912 neurons of the flattened layer. Finally, it is passed through dense and dropout layers. After passing it through a single neuron, the CT scan images are classified as COVID-19 positive or negative [17]. The structure of CTnet-10 is shown in Figure 3.

Narin et al. [18] performed three different CNNs for COVID-19 patient classification in chest X-ray radiographs. ResNet50, InceptionV3, and Inception-ResNetV2 are three different used CNNs in [18]. The reported results show that the pretrained ResNet50 model provides the highest accuracy in comparison with two other CNNs. The used dataset in [18] is composed using fifty COVID-19 patients (from the open source GitHub) and fifty healthy patient's samples (from the Kaggle repository). Although the method presented in [18] provides high accuracy, it has limitations that reduce its use with the same accuracy in a real-world application. The experiments in the [18] were conducted in late 2020 (the time of the beginning of the COVID-19 pandemic). Therefore, the variety of COVID-19 variants in this database is very small. Also, the number of images of patients and healthy people in this paper is considered the same. However, the ratio of patient persons to healthy people is much lower in real conditions and the actual class samples are not balanced. Therefore, teaching deep network with an equal ratio between two classes in this field is not correct. Also, the total number of images is only 100, which is very small.

A deep learning-based approach is proposed by Mahmoudi et al. [19] for COVID-19 detection using CT-imaging. First of all, adaptive histogram equalization is used to reduce noise as preprocess. Next, black slices are removed to crop ROI containing the lungs. Next, a deep U-net is performed based on the CNN encoder and CNN decoder. Finally, a CNN with three layers, with additional fully connected layers followed by a Softmax layer, is used for classification [19]. In [19], the input image is first segmented to separate the region of interest (ROI), including the lungs, from the black areas in the chest CT scan image. Adding the segmentation step increases the computational complexity of the method, compared to other deep-based methods in this area. The ratio of the black area to the lung area is almost constant in CT scans. Therefore, most statistical methods are not sensitive to the size of the ROI. Also, features that are extracted from the ROI are not affected by black areas. Therefore, it is not necessary to use this step.

Some studies employed handcrafted features such as texture and color for COVID-19 recognition. Pereira et al. proposed a classification schema based on the combination of multiclass classification and hierarchical classification [20]. They structured pneumonia as a hierarchy tree, where one of the end nodes is COVID-19. Because of imbalanced data in this domain, they used a resampling algorithm in the proposed schema to rebalance the classes' distribution. Pereira et al. performed some handcrafted texture descriptors such as local binary patterns (LBP), local phase quantization (LQP), local directional number (LDN), and elongated quinary patterns (EQP) to describe chest X-ray images. They explored early and late fusion techniques in order to leverage the strength of multiple texture descriptors and base classifiers [20].

**Figure 4:**
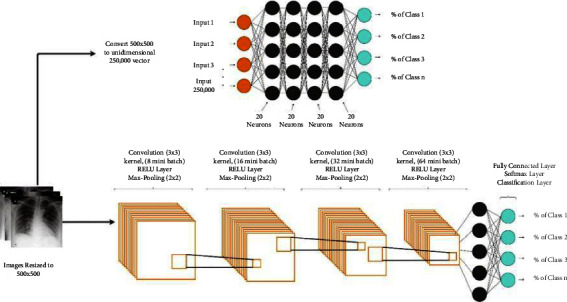
The structure of the CNN and feed forward NN is proposed in [21].

Varela-Santos et al. [21], proposed a supervised learning model to classify COVID-19 patients and some other popular related diseases affecting the lungs. In this respect, statistical texture descriptors such as gray-level co-occurrence matrixes (GLCM) and basic LBP are performed in the feature extraction phase. Six statistical features are extracted using GLCM in different orientations in the joint of a histogram of basic LBP values. Also, two different supervised learning models, feed-forward neural network (FFNN) and CNN are used in the classification phase. The structure of the networks used in [21] for the classification phase is shown in Figure 4.

The results reported in [21], show that the CNN provides higher accuracy than the feed-forward multi-layer network (about 2 percent) on the tested database.

The main goal of [22] is to propose an accurate model for classifying COVID-19 infection in CT scan slices (CTS). The proposed approach in [22] implements a sequence of substeps such as multi-thresholding, image segmentation, feature extraction, feature selection, feature fusion, and classification. The chaotic bat algorithm is used to enhance the CTS contrast. The threshold filter is used to segment the CT scan sample into two segments. The texture features of these images are extracted using discrete wavelet transform (DWT), GLCM, and hu-moments (Hu). Finally, different classifiers such as Naive Bayes (NB), k-Nearest neighbors (KNN), decision tree (DT), random forest (RF), and support vector machine are performed for two-class classification.

Irfan et al. [23] used a combination of tomography CT and chest X-ray to train a hybrid deep neural network model. The proposed approach classified the input samples into three classes namely normal, pneumonia, and COVID-19 patients. Reported results demonstrate high accuracy in comparison with most related methods [23]. Almalki et al. [24] proposed a novel deep network called CoVIRNet for COVID-19 diagnosis. The proposed method in [24] has different inception residual blocks that cater to information at different scales. The features are concatenated at different classification blocks, using the average-pooling layer. Reported results in [24] show that CoVIRNet as a feature extractor in joint of random forest provide higher accuracy than compared methods. Rehman et al. [25], proposed a self-activated convolution neural network for chest diseases classification such as COVID-19. An innovative framework is proposed in for the detection of 15 types of chest diseases, such as COVID-19 disease, via a chest X-ray modality. Firstly, a deep CNN with a soft-max classifier is performed. Next, transfer learning is applied to extract deep features. Finally, deep features are fed to the classical machine learning-based classifiers. The proposed framework improves the accuracy for COVID-19 and predictability rates for other chest diseases in comparison with compared studies [25].

Saeed et al. [26] proposed a mathematical-based method based complex fuzzy hyper-soft set, which is a formation of the complex fuzzy (CF) set and the hyper-soft set. The proposed new fuzzylike hybrid theory provides access to a broad spectrum of membership function values and categorizes the distinct attribute into corresponding subvalued sets [26]. For the COVID-19 variants, a table is constructed relying on the fuzzy interval of [0, 1], which can be used to identify the disease and selects the optimum medication correctly [26]. Allioui et al. [27] proposed a multiagent deep learning-based method for COVID-19 CT scan image segmentation. A new mask extraction approach based on multiagent deep reinforcement learning is presented in [27], to enhance medical image segmentation frameworks. An improved version of the Deep Q-Network is developed to enable the mask detector to choose the best masks from trained images. The proposed approach is used to extract high-level features of COVID-19 infected areas. The reported results show the improvement of the diagnosis accuracy of Covid-19 by using DRL to extract CT masks [27].

## 3. Materials and Methods

**Figure 5:**
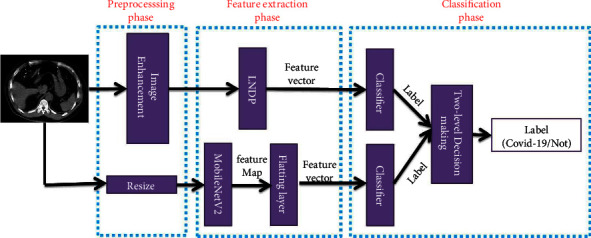
Main block-diagram of proposed approach.

The main aim of this paper is to propose an innovative approach for COVID-19 disease diagnosis in chest CT-scan images based on the combination of low-level and high-level features. In this respect, a multistage approach is proposed that is shown in Figure 5 as block diagram. The proposed recognition approach includes three phases, preprocessing, feature extraction, and classification. The sub-blocks of each phase are described.

### 3.1. Preprocessing Phase

COVID-19 disease has been declared a global pandemic by the WHO since December 2020. Therefore, in almost all countries of the world, chest imaging of suspects is performed to diagnose patients with COVID-19. The technology of different imaging devices, different providers, and technicians' skills in working with the device are not the same in laboratories. Therefore, the method presented in this paper should have a general aspect and have acceptable performance on all images with any quality. Therefore, the image quality must be enhanced before the feature extraction phase. In this step, the histogram equalization algorithm will be performed for each color channel separately.

The preprocessing step consists of image enhancement, noise reduction, and image resizing. As mentioned above, first of all histogram equalization technique is used to enhance the image quality which is efficient in improving the contrast and making the medical image more informative.

Let *f* be a given image represented as a *N*_*r*_ by *N*_*c*_ matrix of integer pixel intensities ranging from 0 to *L* − 1. Let *P* denote the normalized histogram of *f*. So, *P*_*n*_ shows the occurrence probability of intensity *n* in the *f*. The histogram equalized image *g* at position (*i*, *j*), will be defined as follows:

(1) $$\begin{array}{c} G_{i,j}=floor\left(\left(L-1\right)\overset{f_{i,j}}{\underset{n=0}{\sum }}P_{\;n}\right.\text{.} \end{array}$$

The common noises present in chest CT scan are salt & pepper noise, speckles, and impulse noises. Also, a nonlinear median filter in size of 5 × 5, is applied to remove the salt and pepper noise and reduce the impulse noise. A median filter is a nonlinear filter in which each output sample is computed as the median value of the input samples under the considered window, which is in size of 5 × 5 in this paper. That is, the result is the middle value after the input values have been sorted.

MobileNetV2 [28] is very similar to the original MobileNet. It has a drastically lower parameter count than the original MobileNet. MobileNetV2 supports any input size greater than 32 × 32, with larger image sizes offering better performance [28]. So, all of the input images are resized to the acceptable and same size in preprocessing phase.

### 3.2. Feature Extraction Phase

As mentioned above, two sets of features are extracted in this paper to classify chest CT scan images. Instead of most pattern classification approaches, in this manuscript, the feature fusion is not performed in the feature extraction phase and decision fusion is performed in the classification phase, which is one of the main novelties of our proposed approach. So, in the feature extraction phase, low-level features are extracted using LNDP. Also, high-level features are extracted using MobileNetV2.

#### 3.2.1. Texture Low-Level Features

**Figure 6:**
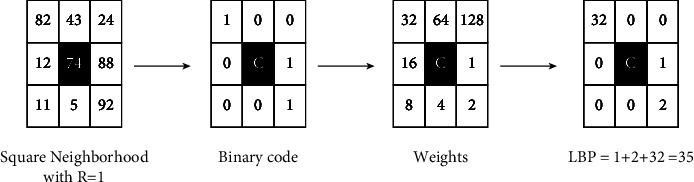
An example of LBP_8,1_ computing process.

Timo Ojala et al. [29] proposed a local texture image descriptor called LBP in 2000. LBP extracts local contrast and local textural spatial structure of the image in case of a histogram of predefined patterns. To calculate LBP at a given pixel coordinate (*x*_*c*,_*y*_*c*_), the local pattern is described as an ordered set of binary comparisons of intensities between the center pixel and its neighbors. In most cases, neighborhood structure is assumed circular to decrease sensitivity to possible image rotations. LBP is defined as follows:

(2) $$\begin{array}{c} LBP_{P,R}=\overset{P-1}{\underset{k=0}{\sum }}\Omega \left(f_{k}-f_{c}\right)2^{k}\text{,} \end{array}$$

(3) $$\begin{array}{c} \Omega \left(x\right)=\left\{\begin{array}{cc} 1\text{,} & ifx\geq 0\text{,}\\ 0\text{,} & else\text{,} \end{array}\right. \end{array}$$

where *f*_*c*_ corresponds to the intensity value of the center, *f*_*k*_ shows the intensity value of the *k*^th^ neighbors and, *P* is the number of neighbors. An example of the LBP computation process is shown in Figure 6. The LBP_*P*,*R*_ descriptor produces (2^*P*^) different binary output values.

**Figure 7:**
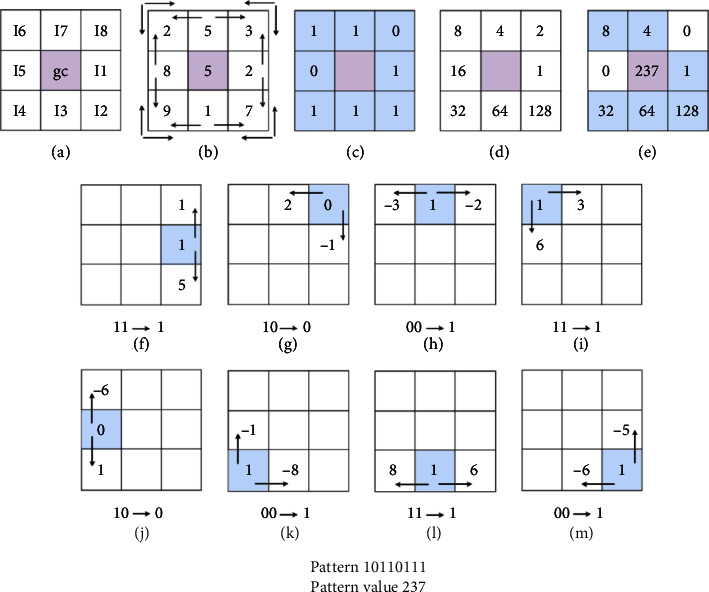
An example of LNDP computing process.

Since then many different extended versions of LBP have been proposed. For example, a one-dimensional local binary pattern (1DBP) is proposed in [30], to reduce the computational complexity of two-dimensional LBP. As another version, the modified local binary pattern is proposed by Ojala et al. [31] to reduce the number of extracted features. Local ternary patterns are proposed in [32] to reduce the noise sensitivity of basic LBP and MLBP. Fekri-Ershad et al. [33] proposed multithreshold uniform local ternary patterns (MT-ULTP) to classify cell phenotypes in fluorescence microscope images. As a new discriminate LBP-like texture descriptors, LNDP was first introduced by Verma et al.in 2017 [34]. The main difference between LNDP and LBP is in neighborhood definition. In LNDP, the internal relation between neighbors is used to describe local texture patterns. In LNDP, for each central pixel like gc, a square block (with radius *r*) around it, is considered as a neighborhood. Next, the following process is performed step by step:

(I) Each neighboring pixel is compared to two adjacent pixels based on equation (4) (the neighboring pixel is defined as a vertical or horizontal pixel).
  (4) $$\begin{array}{c} k_{1}^{n}=\left\{\begin{array}{cc} I_{8}-I_{n}\text{,} & forn=1\text{,}\\ I_{n-1}-I_{n}\text{,} & \forall n=2,3,\ldots ,7\\ I_{n-1}-I_{n}\text{,} & for\;n=8\text{,} \end{array}\right.\text{,}\\ and\text{,}\\ k_{2}^{n}=\left\{\begin{array}{cc} I_{n+1}-I_{n}\text{,} & for\;n\;=1\text{,}\\ I_{n+1}-I_{n}\text{,} & \forall n=2,3,\ldots ,7\text{,}\\ I_{1}-I_{n}\text{,} & for\;n\;=8\text{,} \end{array}\right. \end{array}$$
where the two-by-two relationships of neighbors adjacent to the central *g*_*c*_ pixel are defined according to equation (5). In this regard, *k*_1_^*n*^ and و *k*_2_^*n*^ are similar pairs of neighbors that are adjacent to the central pixel *g*_*c*_, which is the relationship of neighboring pixels for *n* = 1, 2,…, 8. The difference between each neighboring pixel and its two neighbors are shown as *k*_1_^*n*^ and *k*_2_^*n*^. This value is mapped to a corresponding binary number according to equation (5).
(II) The value of each pixel is then mapped to a binary pattern according to equation (5). As a result, a binary pattern is obtained in this step.
  (5) $$\begin{array}{c} S\left(k_{1}^{n},k_{2}^{n}\right)=\left\{\begin{array}{cc} 1, & ifk_{1}^{n}\geq 0\;and\;k_{2}^{n}\geq 0,\\ 1, & ifk_{1}^{n}<0\;and\;k_{2}^{n}<0,\\ 0, & ifk_{1}^{n}\geq 0\;and\;k_{2}^{n}<0,\\ 0, & ifk_{1}^{n}<0\;and\;k_{2}^{n}\geq 0. \end{array}\right.\begin{array}{c} \;\\ \;\\ \; \end{array} \end{array}$$
(III) Extracted binary pattern is multiplied by the corresponding weights to obtain the LNDP value in decimal format.
  (6) $$\begin{array}{c} LNDP\left(g_{C}\right)=\overset{8}{\underset{n=1}{\sum }}2^{n-1}\times S\left(k_{1}^{n},k_{2}^{n}\right)\text{.} \end{array}$$
(IV) Finally, a histogram of LNDP values, which is extracted for each central pixel, is constructed.
(V) The Local Neighborhood Difference (LNDP) descriptor is a statistical image descriptor. The bin values of the normalized extracted histogram is used as a feature vector to describe the texture information of the input image ()
  (7) $$\begin{array}{c} F_{i}=\overset{m-1}{\underset{x=0}{\sum }}\overset{n-1}{\underset{y=0}{\sum }}\left\{\begin{array}{cc} 1,if\;LNDP\left(x,y\right)=i; & i\in \left[0\;,\left(-1\right)\right],\\ 0, & otherwise, \end{array}\right. \end{array}$$
where the number of neighbors is *P*. Also, *M* and *N* show the image's size.  Figure 7 shows an example of the LNDP computation process. The *F*_*i*_ means the *i*^th^ feature value in the final feature vector.

#### 3.2.2. Deep High-Level Features

MobileNet is a type of convolutional neural network designed for mobile and embedded vision applications. They are based on a streamlined architecture that uses depthwise separable convolutions to build lightweight deep neural networks that can have low latency for embedded devices in different scopes such as medical diagnosis. In some recent research, MobileNetV2 has provided acceptable accuracy for medical applications and visual pattern classification. The main goal of this paper was to combine texture information and deep features for COVID-19 diagnosis. In some recent research studies, the MobileNetV2 provided high accuracy on fully textured databases such as Brodatz. This was another reason for choosing this network.

MobileNetV2 is a new deep network that is effective for feature extraction, especially for pattern classification and segmentation [29]. MobileNetV2 is developed by the Google team for the first time. It provides real-time classification capabilities under computing constraints in devices like smartphones. Emotions classification from facial images is commonly used in systems such as user interfaces to enhance the quality of service delivery. According to statistics published by the WHO, early diagnosis of COVID-19 disease can prevent the spread of the disease among other people in the community. Therefore, using MobileNetV2 is a very good option for a real-time recognition system.

**Figure 8:**
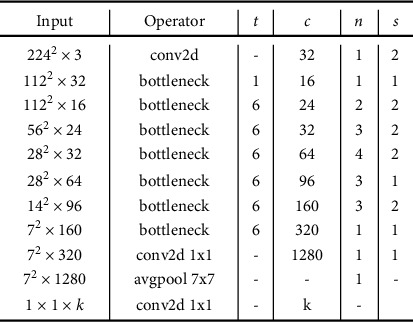
The internal layer's details of MobileNetV2.

MobileNetV2 has a CNN architecture. It is based on an inverted residual structure where the residual connections are between the bottleneck layers. The structure of MobileNetV2 is shown in Figure 8. MobileNetV2 has 53 convolution layers and just one Average Pooling. The MobileNetV2 has two main components in difference from other CNNs: inverted residual block (IRB) and bottleneck residual block (BRB). All of the convolution layers in MobileNetV2 architecture are in one the two following types:

- 1 × 1 convolution
- 3 × 3 depth-wise convolution

**Figure 9:**
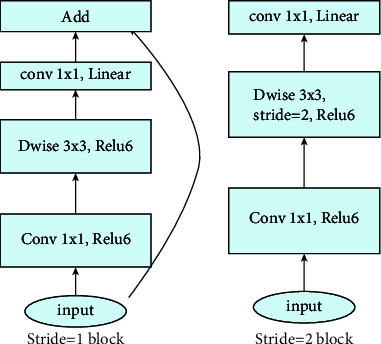
The details of stride 1 block and stride 2 block in MobileNetV2.

There are two different internal blocks named: stride 1 blocks and stride 2 blocks (Figure 9). The internal components of these two block types are shown in Figure 6. The structure of the input layers of the MobileNetV2 is shown in Figure 9. Each line describes a sequence of one or more residual layers, which is repeated *n* times. All layers in the same sequence have the same number of *c* output channels. The first layer of each sequence has a stride *S* and all others use stride 1.

As can be seen in most deep CNNs, the output of each convolution layer is considered as a feature map. As the number of layers increases, the extraction feature maps are deepen and define properties of the image that are not necessarily extractable by the low-level texture descriptors. One of the main innovations of this paper is the combination of low-level and high-level information in the decision-phase. For this purpose, the entire fully connected layer is removed at the end of MobileNetV2 and the prefinal feature map, at the output of the last convolution layer, enters to the proposed flattened layer. Then the output of the flattened layer is used as the feature vector of the desired image representation in the classification phase.

In order to tune hyper-parameters, Adam is performed as an optimizer for 200 epochs with a warm-up learning rate to 0.7 and decays it by 0.1 in epochs 50, 100, and 150. Also, a batch size of 32 for CPU training is used. The current problem in this study is a two class classification, so, in the optimization process, binary cross-entropy is used as a loss function.

### 3.3. Proposed Decision Making Phase

In this paper, for the first time, a two-step algorithm for COVID-19 diagnosis in chest CT-scan images is presented. The results of our research show that the concatenating of texture and deep features does not provide fine accuracy. For this purpose, two separate feature vectors are generated for each image. The first vector contains the features extracted from the image texture using LNDP. The second feature vector includes high-level features that are extracted from the prefinal layer in the MobileNetV2. The test image is then classified based on each one of the feature vectors separately. Each classifier announces a class as the winning class. In this step, the final classification is performed based on the following equation:

(8) $$\begin{array}{c} CF\left(test\right)=\left\{\begin{array}{cc} C_{D}\left(test\right)\text{,} & if\;C_{T}\left(test\right)=C_{D}\left(test\right)\text{,}\\ C_{DM}\left(test\right)\text{,} & else\text{,} \end{array}\right. \end{array}$$

where *E* (test) is the final selected class for test image. *C*_*T*_ (test) is a class selected using the classifier based on texture features. *C*_*D*_ (test) is a class selected using the classifier based on deep features. As can be seen in equation (8), if the label selected by the two classifiers is the same, the same label will be selected as the final result. Otherwise, one of the labels in the second step will be selected based on the following equation:

(9) $$\begin{array}{c} C_{DM}\left(test\right)=\left\{\begin{array}{cc} C_{T}\left(test\right)\text{,} & if\text{,}\;S\left(F_{T}\left(test\right),F_{avg}\left(C_{T}\left(test\right)\right)\right)>\;S\left(F_{D}\left(test\right),F_{avg}\left(C_{D}\left(test\right)\right)\right),\\ C_{D}\left(test\right)\text{,} & if\text{,}\;S\left(F_{T}\left(test\right),F_{avg}\left(C_{T}\left(test\right)\right)\right)<\;S\left(F_{D}\left(test\right),F_{avg}\left(C_{D}\left(test\right)\right)\right)\text{,} \end{array}\right. \end{array}$$

where *F*_*T*_ (test) and *F*_*D*_ (test) are feature vectors extracted for test images based on texture features and deep features. The function *S* (*a*, *b*) shows the similarity value between feature vectors *a* and *b*.

As mentioned in equations (9) & (10), if the label selected by the classifier based on texture features is the same as the label selected by the classifier for deep features, this class is selected as the final class of the test image. If the selected labels based on the two feature groups are not the same, the similarity between the test image and the average sample of both classes in the train set is calculated. Any class that is more similar to the test image is selected as the final class of that image. The mean value in each dimension of all class samples can be considered as the average sample of class.

## 4. Experimental Results

### 4.1. Dataset

**Figure 10:**
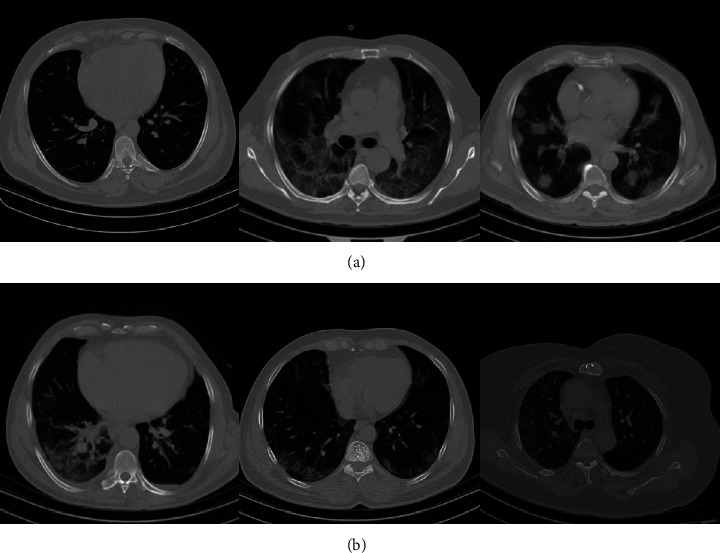
Some chest CT scan samples of the collected database in different scales, (a) covid-19 patients and (b) noncovid cases.

The dataset collection includes chest CT scan images. COVID-19 infected and noninfected or healthy are the two classes into which the data is divided. The CT scan images of COVID-19 patients admitted with a definitive diagnosis of COVID-19 and noninfected patients from June 1, 2021, to December 20, 2021, were extracted from the Picture Archiving and Communication system (PACS) in Ayatollah Taleghani Hospital of Abadan, Iran. The dataset consisted of 238 images. The number of healthy and COVID-19 infected images is equal to 108 and 130, respectively. There is no restriction on the selection of the disease variant, and images of all Covid-19's variants have been collected in this database. This study was approved by the ethical committee board of Abadan University of Medical Sciences (Ethics code: IR.ABADANUMS.REC.1399.207). Some chest Ct-scan examples of the collected database are shown in Figure 10.

### 4.2. Performance Evaluation Metrics

The main aim of this paper is to propose an efficient approach for COVID-19 diagnosis based on chest CT-scan image analysis. So, this problem can be categorized as a visual binary pattern classification problem. Hence, following parameters are considered to evaluate the performance:

(10) $$\begin{array}{c} Accuracy=\frac{TP+TN}{TP+TN+FN+FP}\text{,} \end{array}$$

where TP means true positive, TN means true negative, FP means false positive and FN is a false negative. The current problem is a binary classification, so, true positive means the number of samples who are really patients with COVID-19 and are detected as patients with our proposed system.

As mentioned above, the current problem is a binary classification. Because the transmission frequency of COVID-19 virus is high, the risk of misdiagnosis of a sick person as a healthy person is much higher than the risk of misdiagnosis of a healthy person as a sick sample. Therefore, in addition to the accuracy criterion, the precision measure is evaluated the efficiency of the proposed method. The precision measure focuses on the true positive samples (correct diagnosis of patients).

(11) $$\begin{array}{c} Precision=\frac{TP}{TP+FP}\text{.} \end{array}$$

### 4.3. Performance Evaluation of the Proposed Approach

As mentioned above, a decision-making phase is performed in this paper to improve the final accuracy. Our proposed decision-making algorithm use distance measure to classify label of challengable samples. So, it is possible to perform different distance measures to evaluate the performance of our proposed approach. Also, we performed different efficient classifiers such as k-nearest neighbor (KNN), random forest, and naïve Bayes. The performance of our proposed COVID-19 diagnosis approach is evaluated in terms of accuracy and precision. The results are reported in Table 1 as follows.

**Figure 11:**
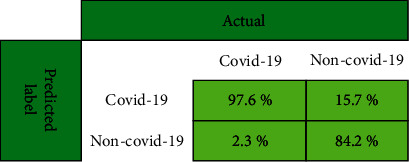
The confusion matrix of our proposed approach in terms of accuracy (%).

As can be seen in Table 1, the proposed approach provide the highest accuracy rate using 5-NN as a classifier based on the cosine similarity measure. The related confusion matrix is reported in Figure 10. The true positive rate of our proposed approach is higher than the true negative rate. Due to the high spread rate of covid-19, the risk of misdiagnosing a person with covid-19 as a healthy person is much higher than the risk of misdiagnosing a healthy person as a patient with covid-19. As you can see in Figure 11, our presented method correctly diagnoses the affected person with a probability of more than 97%. Therefore, the presented method can help to prevent the rate of disease transmission in medical centers.

### 4.4. Comparison with State-of-the-Art Methods

The performance of the proposed approach is compared with some well-known methods in the same situation in terms of validation technique and dataset. The comparison results are shown in Table 2. As can be seen in the first three columns of Table 2 (LNDP + KNN, LNDP + RF, and MobileNetV2), a combination of texture and deep features can provide better performance than using each feature set separately. Results demonstrate that the proposed approach provides higher accuracy in comparison with some state-of-the-art methods in this scope.

In this article, the combination of texture information and deep features extracted from the mobileNetV2 was used to diagnose COVID-19 patients. Therefore, in the results section, we tried to show that the combination of texture features and deep features can provide more accuracy than the use of each of these feature sets separately. Therefore, in Table 2, the diagnosis of COVID-19 patients was performed based on the LNDP operator and with two different classifiers (KNN and RF), and the results were presented. Also, patients were diagnosed once based on two deep MobileNetV2 and ResNet50 and the results were presented in Table 2. Therefore, the lines that are not referenced in Table 2 means that they were implemented by the authors of this article. The comparative results show that the combined mode can provide higher accuracy compared to the use of texture features or deep networks separately.

## 5. Conclusion

The main purpose of this paper was to provide an efficient method for classifying patients with COVID-19 using chest CT-scan images. In this regard, a three-phases method including preprocessing, feature extraction, and classification was presented. In the feature extraction phase, combination of low and high level features are used. LNDP is used to extract low-level texture features. The mobileNetV2 is used to extract high-level deep features, and by removing the last softmax layer, deep features were creatively extracted. Also, for the classification phase, an innovative method based on the output combination of different classifiers is proposed to decide on challenging samples. The efficiency of the proposed method is evaluated on a database of chest CT scan images of patients and healthy individuals in Medical Abadan Hospital. The results showed that the proposed method provides good accuracy for diagnosing COVID-19 disease compared to many existing methods.

The proposed approach (CTFDF) provides 91.61 percent accuracy using 5-NN as a classifier based on cosine similarity. Due to the high spread rate of COVID-19, the risk of misdiagnosing a person with COVID-19 as a healthy person is much higher than in other situations. CTFDF correctly diagnoses the affected person with a probability of more than 97%. The true positive rate of our proposed approach is higher than the true negative rate about 13.4 percent. The performance of the proposed approach is compared with some well-known methods in this scope. In this article, the combination of texture information and deep features extracted from the mobileNetV2 was used to diagnose COVID-19 patients. The results showed that the presented combined method provides 1.57 percent more accuracy than the original mobileNetV2 and 7.02% more accuracy than the used LNDP texture features. Low sensitivity to noise due to the use of global statistical features and deep features are other advantages of the proposed method. The results show that the combination of texture and deep features can provide better performance than using each feature set separately. Results demonstrate that the proposed approach provides higher accuracy in comparison with some state-of-the-art methods in this scope.

In this paper, a creative method for extracting deep features from the mobileNetV2 and combining with texture information is presented. The proposed method is not problem-dependent. So as the future work idea, the proposed feature extraction method can be used in many other common computer vision problems in the learning phase such as cervical cancer diagnosis *r* lung cancer detection. To increase the accuracy of challenging sample classification, a two-step decision-making method is proposed for the first time that has a general aspect and can be applied to other issues in the machine learning area.

As mentioned in the text, the proposed algorithm only pays attention to the predicted labels in previous steps. So, it is independent form the determining methods in the previous steps. Therefore, the proposed algorithm is a general method, which can be used in various problems in the field of supervised machine learning to improve the final accuracy, especially in hybrid classifiers. In this article, the main structure of the layers in the MobileNetV2 has not changed, so as future works, the structure of the layers, the number of layers, and other parameters of the network can be optimized. Therefore, the use of these two proposed innovative methods in other problems can be suggested as future works. The number of features extracted from a chest CT scan image in this paper is greater than a simple deep network such as a mobileNetV2. This is one of the minor limitations of the proposed method. However, due to the removal of the softmax and final max-pooling layers and the use of two simple linear statistical classifiers, the total computational complexity of the proposed method is in the same range compared to deep networks.

## Figures and Tables

**Table 1:** Performance evaluation results of the proposed approach in terms of accuracy (%) and precision (%).

Distance measure	Classifier						
	Measure	3-NN	5-NN	7-NN	Random forest (trees = 100)	Random forest (trees = 150)	Naïve Bayes
Euclidean	Accuracy	87.02	90.02	89.49	86.88	84.93	80.17
	Precison	88.27	90.54	89.92	86.77	85.64	80.67

Cosine	Accuracy	88.23	91.61	91.27	86.38	85.26	81.29
	Precison	88.65	91.79	91.18	86.89	85.17	82.04

Log-likelihood	Accuracy	88.68	91.09	90.56	87.01	85.87	82.37
	Precison	89.04	91.82	90.77	87.33	85.94	82.65

**Table 2:** Comparision results with state-of-the-art methods in terms of accuracy (%).

Approach	Accuracy
LNDP + KNN (base line)	84.59
LNDP + RF (base line)	82.25
MobileNetV2 (base line)	90.04
ResNet50 (base line)	90.17
VGG-19 [35]	87.93
SVM + LBP [21]	87.54
CNN + LBP + GLCM [22]	88.39
CTFDF (proposed method)	91.61

## Data Availability

The data used to support the findings of this study are available from the corresponding author upon request.

## References

[1] worldometers.info (2022). COVID-19 CORONAVIRUS PANDEMIC. https://www.worldometers.info/coronavirus.

[2] Filice R. W. (2021). *The Imaging Informatics Response to a Pandemic*.

[3] Ghaderzadeh M., Asadi F., Jafari R., Bashash D., Abolghasemi H., Aria M. (2021). Deep convolutional neural network–based computer-aided detection system for covid-19 using multiple lung scans: design and implementation study. *Journal of Medical Internet Research*.

[4] Wang W., Xu Y., Gao R. (2020). Detection of SARS-CoV-2 in different types of clinical specimens. *Jama*.

[5] Long C., Xu H., Shen Q. (2020). Diagnosis of the Coronavirus disease (COVID-19): rRT-PCR or CT?. *European Journal of Radiology*.

[6] Shi W., Tong L., Zhu Y., Wang M. D. (2021). COVID-19 automatic diagnosis with radiographic imaging: explainable attention transfer deep neural networks. *IEEE Journal of Biomedical and Health Informatics*.

[7] Ai T., Yang Z., Hou H. (2020). Correlation of chest CT and RT-PCR testing for coronavirus disease 2019 (COVID-19) in China: a report of 1014 cases. *Radiology*.

[8] Fang Y., Zhang H., Xie J. (2020). Sensitivity of chest CT for COVID-19: comparison to RT-PCR. *Radiology*.

[9] Shi F., Wang J., Shi J. (2021). Review of artificial intelligence techniques in imaging data acquisition, segmentation, and diagnosis for COVID-19. *IEEE Reviews in Biomedical Engineering*.

[10] Wang S., Kang B., Ma J. (2021). A deep learning algorithm using CT images to screen for Corona Virus Disease (COVID-19). *European Radiology*.

[11] Tsai E. B., Simpson S., Lungren M. P. (2021). The RSNA international COVID-19 open radiology database (RICORD). *Radiology*.

[12] Jungmann F., Müller L., Hahn F. (2022). Commercial AI solutions in detecting COVID-19 pneumonia in chest CT: not yet ready for clinical implementation?. *European Radiology*.

[13] Aria M., Nourani E., Golzari Oskouei A. (2022). ADA-COVID: adversarial deep domain adaptation-based diagnosis of COVID-19 from lung CT scans using triplet embeddings. *Computational Intelligence and Neuroscience*.

[14] He X., Yang X., Zhang S., Zhao J., Zhang Y., Xing E. (2020). Sample-efficient deep learning for COVID-19 diagnosis based on CT scans. *Medrxiv*.

[15] Alshazly H., Linse C., Barth E., Martinetz T. (2021). Explainable COVID-19 detection using chest CT scans and deep learning. *Sensors*.

[16] Khan A. I., Shah J. L., Bhat M. M. (2020). CoroNet: a deep neural network for detection and diagnosis of COVID-19 from chest x-ray images. *Computer Methods and Programs in Biomedicine*.

[17] Shah V., Keniya R., Shridharani A., Punjabi M., Shah J., Mehendale N. (2021). Diagnosis of COVID-19 using CT scan images and deep learning techniques. *Emergency Radiology*.

[18] Narin A., Kaya C., Pamuk Z. (2021). Automatic detection of coronavirus disease (covid-19) using x-ray images and deep convolutional neural networks. *Pattern Analysis and Applications*.

[19] Mahmoudi R., Benameur N., Mabrouk R., Mohammed M. A., Garcia-Zapirain B., Bedoui M. H. (2022). A deep learning-based diagnosis system for COVID-19 detection and pneumonia screening using CT imaging. *Applied Sciences*.

[20] Pereira R. M., Bertolini D., Teixeira L. O., Silla C. N., Costa Y. M. (2020). COVID-19 identification in chest X-ray images on flat and hierarchical classification scenarios. *Computer Methods and Programs in Biomedicine*.

[21] Varela-Santos S., Melin P. (2021). A new approach for classifying coronavirus COVID-19 based on its manifestation on chest X-rays using texture features and neural networks. *Information Sciences*.

[22] Kadry S., Rajinikanth V., Rho S., Raja N. S. M., Rao V. S., Thanaraj K. P. (2020). Development of a machine-learning system to classify lung CT scan images into normal/COVID-19 class. http://arXiv.org/abs/2004.13122.

[23] Irfan M., Iftikhar M. A., Yasin S. (2021). Role of hybrid deep neural networks (HDNNs), computed tomography, and chest X-rays for the detection of COVID-19. *International Journal of Environmental Research and Public Health*.

[24] Almalki Y. E., Qayyum A., Irfan M. (2021). A novel method for COVID-19 diagnosis using artificial intelligence in chest X-ray images. *Healthcare*.

[25] Rehman N. U., Zia M. S., Meraj T. (2021). A self-activated cnn approach for multi-class chest-related COVID-19 detection. *Applied Sciences*.

[26] Saeed M., Ahsan M., Saeed M. H. (2022). An optimized decision support model for COVID-19 diagnostics based on complex fuzzy hypersoft mapping. *Mathematics*.

[27] Allioui H., Mohammed M. A., Benameur N. (2022). A multi-agent deep reinforcement learning approach for enhancement of COVID-19 CT image segmentation. *Journal of Personalized Medicine*.

[28] Sandler M., Howard A., Zhu M., Zhmoginov A., Chen L. C. Mobilenetv2: inverted residuals and linear bottlenecks.

[29] Ojala T., Pietikäinen M., Mäenpää T. Gray scale and rotation invariant texture classification with local binary patterns.

[30] Tajeripour F., Fekri-Ershad S. (2014). Developing a novel approach for stone porosity computing using modified local binary patterns and single scale retinex. *Arabian Journal for Science and Engineering*.

[31] Ojala T., Pietikainen M., Maenpaa T. (2002). Multiresolution gray-scale and rotation invariant texture classification with local binary patterns. *IEEE Transactions on Pattern Analysis and Machine Intelligence*.

[32] Tan X., Triggs B. (2010). Enhanced local texture feature sets for face recognition under difficult lighting conditions. *IEEE Transactions on Image Processing*.

[33] Fekri-Ershad S. (2021). Cell phenotype classification using multi threshold uniform local ternary patterns in fluorescence microscope images. *Multimedia Tools and Applications*.

[34] Verma M., Raman B. (2018). Local neighborhood difference pattern: a new feature descriptor for natural and texture image retrieval. *Multimedia Tools and Applications*.

[35] Kamil M. Y. (2021). A deep learning framework to detect Covid-19 disease via chest X-ray and CT scan images. *International Journal of Electrical and Computer Engineering (IJECE)*.

